# The recursive grammar of mental time travel

**DOI:** 10.1098/rstb.2023.0412

**Published:** 2024-09-16

**Authors:** Jonathan Redshaw

**Affiliations:** ^1^ The University of Queensland, Brisbane, Australia

**Keywords:** recursion, metarepresentation, memory, prospection, iteration, counterfactual thinking

## Abstract

One apparent feature of mental time travel is the ability to recursively embed temporal perspectives across different times: humans can remember how we anticipated the future and anticipate how we will remember the past. This recursive structure of mental time travel might be formalized in terms of a ‘grammar’ that is reflective of but more general than linguistic notions of absolute and relative tense. Here, I provide a foundation for this grammatical framework, emphasizing a bounded (rather than unbounded) recursive function that supports mental time travel to a limited temporal depth and to actual and possible scenarios. Anticipated counterfactual thinking, for instance, entails three levels of mental time travel to a possible scenario (‘in the future, I will reflect on how my past self could have taken a different future action’) and is centrally implicated in complex human decision-making. This perspective calls for further research into the mechanisms, ontogeny, functions and phylogeny of recursive mental time travel, and revives the question of links with other recursive forms of thinking such as theory of mind.

This article is part of the theme issue ‘Elements of episodic memory: lessons from 40 years of research’.

## 1. Introduction

When you were a child, what did you want to do when you grew up? When you reach the end of your life, how do you think you will look back on what you actually did? Humans can not only mentally travel to the past and future, but also remember our past anticipations and anticipate our future remembrances. We can likewise remember how we once remembered the past, anticipate how we will later anticipate the future, and even anticipate how we will remember our anticipations—and later remember those anticipated remembrances of our anticipations. If you have lost track of all these temporal levels, perhaps consider that if you have ever reflected on a past mistake and thought to yourself ‘I knew I would regret doing that’, then you were essentially remembering (level 4) how you anticipated (level 3) that you would eventually reflect on (level 2) that mistake and wish you had chosen a different future (level 1). At face value, such multi-layered mental time travels are recursive [1,2], in that the cognitive processes responsible for representing a temporal perspective seem to call upon those same cognitive processes to represent another temporal perspective with hierarchical structure.

Linguists have long alluded to a recursive structure of human temporal thought (e.g. [3,4]), demarcating relative tense forms such as past perfect (remembered past-orientation), future perfect (anticipated past-orientation), future in the past (remembered future-orientation), future in the future (anticipated future-orientation) and even future perfect in the past (remembered anticipated past-orientation). Indeed, Corballis [1,5] proposed that such tense forms, which do not appear in all languages, are but one manifestation of a universal human ability to recursively embed past and future minds. Psychologists working directly on mental time travel, nonetheless, typically employ a shallower taxonomy of temporal thought, largely restricting our study to the cognitive equivalents of absolute tense: simple past and simple future. This is not to say that work on recursive mental time travel is entirely absent. There is, for instance, a healthy tradition of research on the cognitive equivalent of conditional perfect tense—counterfactual thinking—in which one imagines what the present would look like had a past contingency been otherwise [6–8]. Yet, with a few notable exceptions (e.g. [9–12]), there has been remarkably little acknowledgement that this form of counterfactual thinking entails mental time travel to an alternative future—from the relative perspective of the absolute past. More generally, what seems to be lacking is an integrative framework of the recursive ‘grammar’ of mental time travel that incorporates both absolute and relative temporal reference.

Here, I lay the groundwork for such a grammatical framework, intending that it may be further developed, refined and formalized in future work. This groundwork is itself inspired by the ideas of Suddendorf and Corballis [1,2,5,13–15], who have previously made cases for the importance of recursive processes in temporal cognition. I nonetheless extend their ideas by delimiting two different forms of recursion in human mental time travel, by distinguishing various expressions of temporal cognition according to a recursive representational hierarchy, and by continuing some recent efforts [10,11,16] to amalgamate work on mental time travel with work on modal cognition (i.e. reasoning over possibilities [17,18]). I also introduce a preliminary bracketed notation by which to classify recursive mental time travel, suggest how and why the recursive depth of mental time travel may be limited in humans, and discuss the potential relationship between recursive mental time travel and theory of mind. In the spirit of an Opinion article, and in the interest of stimulating further debate and research, some of my arguments draw on thought experiments and conjecture. At the end of the article I therefore emphasize the open questions in this area and call for additional conceptual and empirical inquiry.

## 2. In what sense is mental time travel recursive?

While it is evidently possible to delineate a recursive structure of mental time travel, it is less clear just how—or even if—this structure is cognitively substantiated. As has been argued for language (e.g. [19,20]), it is possible that the apparently recursive structure of mental time travel is merely an artefact of the human capacity to recognize and formulate such structure, rather than reflecting any underlying (neuro)cognitive processes that call upon themselves. It may instead be, for instance, that each apparent representational level is substantiated via a distinct cognitive process, or that all concurrently represented levels are somehow ‘chunked’ in a non-hierarchical manner. By contrast, if one gives credence to the language of thought hypothesis—which broadly posits that thought has a syntactical structure analogous to that of language [21,22]—then the simple fact that we can formalize a coherent recursive (i.e. syntactical) structure of mental time travel provides circumstantial evidence for the veracity of that structure (also see [23]). As will become clear, thinking of mental time travel as a recursive process at the very least provides a useful means for cognitive scientists to identify and study various forms of temporal thought both common and rare in humans—both on the strong version of my proposal (mental time travel is a recursive cognitive process) and the weak version of my proposal (mental time travel can be systematically modelled as a recursive process).

Although I cannot hope to resolve the fundamental question of whether mental time travel truly is recursive here, I do hope to strengthen the contention [1,2,13,24,25] that it shares some very suggestive similarities with other processes traditionally conceived of as recursive. In linguistics and computer science, ‘recursion’ has two related but distinct meanings [26,27]. One sense of recursion, *tail recursion*, roughly involves taking a series of complete elements and then adding another element of the same category to either end of the series. Drawing on similar illustrations (e.g. [1,2,20]), here are two equivalent linguistic examples of tail recursion with six elements (i.e. six clauses) each—the first an example of right-tail recursion and the second an example of left-tail recursion:

(i) *The farmer yelled at the dog that barked at the cat that chased the rat that bit the mouse that ate the cheese that sat in the field*.

(ii) *In the field sat the cheese that was eaten by the mouse that was bitten by the rat that was chased by the cat that was barked at by the dog that was yelled at by the farmer*.

The other sense of recursion, *nested recursion* (or centre-embedded recursion), roughly involves partitioning one element of a series and embedding a second element of the same category, such that the second element is called upon and completed before the first element completes.^1^ Here is a grammatically correct linguistic example of nested recursion with six elements, which is equivalent to examples (i) and (ii):

(iii) *The cheese that the mouse that the rat that the cat that the dog that the farmer yelled at barked at chased bit ate sat in the field*.

Online comprehension of sentences with tail recursion, like (i) and (ii), is not bounded by the total number of elements in the series, such that we can first grasp the relation between A and B, and then the relation between B and C, and so on. An equivalent sentence with nested recursion, like (iii), by contrast, can be unintelligible [1].

Mental time travel appears to exhibit analogous online processing constraints as sentences with nested recursive structure. It seems implausible, for instance, that anyone would anticipate remembrance of anticipated anticipation of remembered anticipation of remembered remembrance of anticipation, whereas mere anticipated remembrance of anticipation (e.g. ‘I would regret doing that’) is within our grasp. One possible explanation for these comparable constraints is that higher-level mental time travel entails nesting a representation of one’s own mind from a given time within another representation of one’s own mind from a different time [2,13]. This *representational nesting* need not be episodic in character, such that one might simultaneously represent the episodic perspectives of all the different ‘selves’ at all temporal levels. Rather, the nested structure could be encoded propositionally [23], with one able to mentally attend to and switch between any episodic perspective in a serial manner (see §3 for an elaboration of such propositional encoding). Indeed, although recursive temporal thinking might typically entail an element of episodic cognition, there is no need to presuppose that episodic cognition is a necessary constituent of the process; some expressions of such thinking might exclusively entail semantic memory [28] and semantic future thinking [29] across multiple levels (as when you might recognize an old calendar note about meeting a friend for lunch, without being able to envision either writing the note or meeting the friend).

Notably, previous frameworks of mental time travel have already expounded upon the role of recursive operations in the human capacity for *generative thinking*: the ability to imagine whatever, wherever, whenever [1,14,15,30]. In Suddendorf & Corballis’ [2] theatre metaphor of mental time travel, for instance, the ‘playwright’ populates the mental stage with imagined actors, objects and settings in a discretely infinite manner [31], functioning to enable the unbounded construction of potential future scenarios (also see [32]). However, this notion of recursion is more reflective of tail recursion than nested recursion, in that the elements of generative thought accumulate within a single imagined scenario playing out over time, rather than being hierarchically organized across different scenarios. Indeed, whereas the capacity for generative thinking might be considered to increase the breadth of mental time travel in an unbounded fashion, the capacity for representational nesting might be considered to increase the depth of mental time travel in a bounded fashion. Although here I largely restrict my analysis to representational nesting, future research may wish to more closely examine the relationship between these two apparently recursive features of human mental time travel.

## 3. A recursive representational hierarchy

This section outlines a hierarchy of representations that illustrate the nested recursive structure of mental time travel. As will become clear, each of these representations varies on three pertinent dimensions. First, only some representations are nominally *recursive*, in that the cognitive processes involved in representing a temporal perspective seem to call on those same cognitive processes to represent another temporal perspective with hierarchical structure. Second, these representations vary in *multiplicity*, in that some refer to a series of singular perspectives and events structured across time, whereas others refer to merely possible events with represented or implied alternatives (equivalent to the conditional mood in language). Third, these representations vary in *relative temporal structure*, or the unique structure of past- and future-oriented temporal relations among the encoded perspectives and events (as exemplified by the difference between remembered anticipation and anticipated remembrance).

The section is primarily organized according to the maximum level of recursive nesting entailed in a given representational structure. I commence at level 0, emphasizing that some basic forms of temporal cognition may not be recursive and yet might still have important functions. I then move on to level 1 (encompassing episodic memory and episodic foresight), which is arguably the first recursive level of mental time travel, before progressing through higher and more unambiguously recursive levels. From level 1 onwards, I implement a preliminary bracketed notation to signify the relative temporal relations between the different levels of representational structure, with relatively past-oriented perspectives denoted by {past} and relatively future-oriented perspectives denoted by {future}. In this notation, the level 1 representation—i.e. the only representation that does not call upon another representation—is always denoted in the innermost brackets. A recursive representational structure with three levels might thus take the general form of {level3{level2{level1}}}, as exemplified by {past{future{past}}}. As will be clarified, however, such representational structures might also be considered to incorporate one’s present perspective at the outermost level, taking the general form of {present{level3{level2{level1}}}} and exemplified by {present{past{future{past}}}}. This notation has the advantage of encompassing all cognitive equivalents of relative tense forms (e.g. past perfect and future perfect), alongside forms of temporal thinking that have no tense equivalent in English or any other language. Still, the notation lacks precision in some respects, and I discuss potential revisions towards the end of the article.

### (a) Level 0

Some cognitive processes seem to refer to specific past or future events and yet do not have recursive structure and do not even involve mental time travel. In particular, these cognitive processes lack *autonoesis*—conscious awareness of represented experiences as being located in the past or future—which Tulving [28,33] classically identified as an essential component of episodic cognition. Mind-wandering, for instance, often involves representations of past or potential future events [34], and yet much of mind-wandering occurs without awareness [35]—as when reading a boring passage of text and suddenly realizing that your mind had drifted off while your eyes were still scanning the page. Such involuntary mind-wandering might have originally evolved as a precursor to full-blown mental time travel, functioning to prompt animals to act with reference to past or potential future events without having to represent these events as being located at particular times [36]. Hoerl & McCormack [37] make a similar case for a primordial *temporal updating system*, which might enable an animal to maintain a tenseless representation of its broader environment (i.e. beyond its current sensory scope) and the actions it might take in that environment.

Other neurocognitive processes that appear to refer to past or potential future events without necessarily requiring autonoesis include hippocampal replay and preplay sequences, which map out particular spatial routes in an animal’s immediate [38,39] or broader environment [40,41]. Among rodents—and probably other mammals—replay and preplay sequences correlate with actual past and future behaviour, potentially functioning to consolidate memories and aid navigational decisions [42–44]. These sequences also, however, typically play out around 10 times faster than *in situ* navigation [45], rather than playing out in roughly real time as human mental time travel is often conceived of [46]. Indeed, as Comrie *et al*. [47, p. 11] emphasize, such hippocampal activity ‘can be understood at the level of the brain and need not entail conscious awareness or mental imagery’. It seems unlikely, therefore, that that this activity is essentially autonoetic in and of itself.

Intriguingly, there is some evidence that hippocampal preplay sequences can rapidly cycle between two alternative possible future actions via the theta rhythm [38], and that ‘replay’ sequences often encode counterfactual past actions rather than actual past actions [48,49]. Such representations might function to enable animals to select between and act with reference to mutually exclusive alternatives, without necessarily understanding the mutually exclusive relation that links such alternatives [11,18] or even being consciously aware of each alternative [47]. Indeed, assuming that these representations are not accompanied by autonoesis, it would appear that even rudimentary, non-recursive temporal representations can encode multiplicity. Such level 0 representations of alternative future and counterfactual past possibilities might thus be conceived of as ‘pseudo’ forms of the mental time travel with multiplicity that I cover below.

### (b) Level 1

The bulk of direct work on mental time travel has examined level 1 processes: *episodic memory* [50] and *episodic foresight* [51] (or episodic future-thinking [52]). By definition, these processes entail the autonoetic awareness that one is mentally projecting oneself through time [28,33]. What is less clear, however, is whether level 1 mental time travel qualifies as recursive. At face value, it is non-recursive [2], in that the cognitive processes responsible for generating a mental scenario do not seem to be called upon to generate another mental scenario with hierarchical structure. If, however, autonoesis depends on the metarepresentational understanding that one’s *present* mind can hierarchically represent one’s past or future mind [1,13,36]—thus endowing mental time travel with its temporal quality [36]—then it follows that episodic memory and foresight are indeed recursive with temporal structures of {present{past}} and {present{future}}. Although this distinction might seem trivial, the recursiveness or non-recursiveness of autonoesis could prove one key to progressing the controversy surrounding whether non-human animals are capable of mental time travel [2,37,53–57]. That is, if it was conclusively demonstrated that autonoesis entails recursive cognition, then one might be less inclined to attribute level 1 mental time travel capacities to animals (who show no compelling evidence of recursive communication [31,58]) than if it was demonstrated that autonoesis does not entail recursive cognition. I do not intend to resolve this question one way or the other here, but for the sake of consistency I will continue to incorporate the present mind when denoting relative temporal structure.

Level 1 mental time travel can vary in multiplicity, in that people are not constrained to imagining only one version of an event located at a given time. Multiplicity is often evident in episodic foresight, for instance, when you imagine and compare mutually exclusive versions of a single future event—with a plural temporal structure of {present{futures}}. This process can be as simple as imagining and preparing for two possible paths that an object might take [59,60] or as complex as evaluating several possible career choices you might make. Elsewhere, myself and Suddendorf [11] have argued that an explicit understanding of mutually exclusive futures entails a capacity to represent *temporal junctures*, or points in time where subjectively possible versions of reality diverge from one another like a fork in the road. Nonetheless, it is also common to compare different futures without representing temporal junctures, as when you might compare a utopian future to a dystopian future without necessarily imagining the contingencies that could lead to one or the other. However, because such cases lack a represented temporal relation linking the alternatives (i.e. a temporal juncture), it may be more accurate to conceive of them as entailing multiple distinct representations with singular structure—{present{futureA}} and {present{futureB}}—rather than an integrated representation in the form of {present{futures}}. A similar case has been put forward to explain how young children might conceive of mutually exclusive possibilities, A and B, without necessarily representing the exclusive-OR relation that links them [61].

It is likewise possible to compare level 1 representations of the past without representing temporal junctures, with these representations having distinct singular structures of {present{pastA}} and {present{pastB}}. You might make such a comparison, for instance, when asked to rank two job candidates based on their interview performances. However, because represented temporal junctures inherently face forward with the arrow of time [11], it is not clear that it makes sense to delineate level 1 mental time travel structures with integrated past multiplicity in the form of {present{pasts}}.^2^ Rather, as will be clarified below, representing alternative versions of the past within the same hierarchical structure might necessitate level 2 mental time travel (also see McCormack & Hoerl’s [63] framework of *event-independent time* that proposes a similar asymmetry between future and past).

### (c) Level 2

At the second level and above, the structure of mental time travel is more unambiguously recursive [2], even among instances without multiplicity. Some such instances have a {present{past{past}}} structure, as when recalling what someone else told you about a past event (i.e. hearsay), whereas others have a {present{future{future}}} structure, as when you propose a meeting at which you will plan an upcoming event. Conversely, you might recall what someone else told you about their plan, structured {present{past{future}}}, or propose a meeting to discuss a recent incident, structured {present{future{past}}}. Level 2 mental time travel can also take forms with both recursiveness and multiplicity, like {present{future{futures}}}, {present{futures{future}}} or even {present{futures{futures}}}. The final form, for instance, would encompass a situation where a group must decide between two possible meeting venues (level 2), at which they will discuss which of two possible plans they will enact (level 1). Thus, recursive mental time travel with multiple future-oriented perspectives can incorporate temporal junctures at both higher and lower levels.

Much of the relevant work on level 2 mental time travel has examined *counterfactual thinking*. Although this term has alternative meanings [64–66], one research tradition holds that counterfactual thinking involves mentally undoing an actual past event and imagining what the present would look like had an antecedent of that event unfolded otherwise [6,67,68]. After missing an early morning meeting, for instance, you might think to yourself ‘if only I had set my alarm last night…’ or ‘if only my bus had arrived on time…’. Implicated in this process is the recognition that, in the absolute past, there were alternative ways the relative future could have unfolded [9,10] at a temporal juncture [11]. This form of counterfactual thinking thus has a temporal structure featuring both recursiveness and multiplicity, {present{past{futures}}}.

Counterfactual thinking appears to have many important functions in human cognition and behaviour [18]. Causal judgements about past events, for instance, have been proposed to rely on counterfactual simulation, with experiments showing that adults tend to judge that object X caused event Y if and only if event Y would not have eventuated in the case that object X was absent [69,70]. Counterfactual thinking also supports moral judgements, such that people (including children aged 6 years and older [71,72]) tend to make stronger judgements about a character’s ‘good’ or ‘bad’ past action if that character had a counterfactual choice available to them at the time. People similarly tend to make stronger judgements about their own past actions if there were counterfactual alternatives available at the time [73–75]. The counterfactual emotions of regret [76] and relief [77], for instance, arise when you consider that you could have taken a better or worse past action, respectively, than your actual past action. Accordingly, the functional theory of counterfactual thinking [78,79] suggests that people tend to focus on counterfactuals that were within their own control (e.g. ‘I wish I had brought my umbrella’) rather than counterfactuals that were out of their control (e.g. ‘I wish it hadn’t started raining’), given that only controllable factors can be altered in similar future situations (also see [80]). That is, counterfactual thinking with the level 2 form of {present{past{futures}}} may often function to aid mental time travel with the level 1 form of {present{futures}}.

### (d) Level 3

From level 3 onwards, the range of conceivable temporal structures considerably expands. Some of these structures may be rarely expressed, including the one-way journeys of {present{past{past{past}}}} and {present{future{future{future}}}}. It is nonetheless plausible, for instance, that you would set a reminder in your calendar for tomorrow morning, because you plan (level 3) to contact your colleagues to propose a meeting (level 2) at which you will plan an upcoming event (level 1). Other structures of level 3 mental time travel include {present{past{future{past}}}}, such as when you remember (level 3) that you had planned (level 2) to regale your friend with an entertaining anecdote about a recent past event (level 1)—i.e. ‘I’ve been meaning to tell you about what happened to me the other day’.

Perhaps the most common form of level 3 mental time travel is *anticipated counterfactual thinking*, in which one foresees looking back on a relative past decision and imagining what else one could have done [9,10,81] at the relevant temporal juncture [11]. Anticipated counterfactual thinking can take one of two temporal structures. The first structure, {present{future{past{futures}}}}, entails a situation in which you have already taken a particular course of action, yet you nonetheless recognize that in the future (level 3) you will continue to reflect on that past action (level 2) and consider what would have happened if you had chosen an alternative course of action at the time (level 1). Such thinking might be common, for instance, after making an especially consequential life choice like accepting a job offer or immigrating to a new country—i.e. ‘I will always wonder what my life would have been like if I did not...’. The second structure of anticipated counterfactual thinking, {present{futures{past{futures}}}}, entails a situation in which you have yet to make up your mind about a future course of action, and for at least one possible future action (level 3) you consider how you would eventually reflect on (level 2) that action and its alternatives (level 1).

Anticipated counterfactual thinking with the first structure may function in a similar manner to level 2 counterfactual thinking, in that it may prompt us to make better decisions when confronted with similar future choices with the level 1 form of {present{futures}}. Anticipated counterfactual thinking with the second structure, however, appears much more straightforwardly functional, in that it can prompt us to make a better decision before any such decision has been made. One common domain in which such thinking may prompt better decisions is intertemporal choice, in which an agent is confronted with a decision between a smaller, sooner reward or a larger, later reward [82]. For instance, by considering that you might eventually (level 3) look back with regret (level 2) at choosing the tempting sooner reward (level 1), you may be more likely to take the alternative level 1 action of choosing the later reward (see [83]). One general function of mental time travel is that it enables us to pre-experience the emotional valence of potential future events [84,85], and pre-experiencing regret in particular may provide sufficient motivation to take a particular course of action even when another course of action has more immediate appeal. Accordingly, anticipated regret is a strong predictor of health-related choices [86], including in the domains of exercise [87], nutrition [88] and vaccine uptake [89]. Indeed, whereas the functional theory of counterfactual thinking [78,79] itself has only mixed empirical support [90], the functional value of anticipated counterfactual thinking in the form of {present{futures{past{futures}}}} is almost self-evident.

### (e) Higher levels

Recursive mental time travel beyond level 3 is sometimes feasible [11]. You can probably, for instance, recall a past moment of anticipated counterfactual thinking, with such recollection perhaps taking the level 4 form of {present{past{futures{past{futures}}}}}. If you squint hard enough, you might even be able to imagine how you would recall that moment of anticipated counterfactual thinking tomorrow, with a level 5 form of {present{future{past{futures{past{futures}}}}}}. Alternatively, you might remember the beginning of this article, where I asked you to reflect on a level 4 moment of thinking ‘I knew I would regret doing that’—such that your current remembrance (level 6) of that prompted reflection (level 5) arguably takes the form of {present{past{past{past{futures{past{futures}}}}}}}. Alas, I suspect it might be more tempting to chunk the lower five levels together and simply remember (level 2) that you read such a passage that asked you to reflect on a past moment (level 1).

Evidently, such high levels of mental time travel can be cognitively taxing and impractical. As outlined earlier, the proximate explanation for this cognitive difficulty might involve a limit on the number of nested relations that humans can concurrently entertain. Ultimately, however, there might be little selective pressure to further expand this limit, given that higher limits might have rapidly diminishing returns. Indeed, whereas a soft limit of 3 levels of mental time travel is plainly useful from an evolutionary standpoint—in that it provides the means to pre-experience some delayed emotional consequences of level 1 decisions (via anticipated regret and relief [11])—there is no such obvious gain to be drawn from higher levels. It is even feasible that higher levels of mental time travel could lead to maladaptive decision hesitancy, in that it might be difficult to efficiently resolve the intrapersonal conflict that would arise between more and more versions of the self represented across time. Accordingly, consistent with the Goldilocks principle (e.g. [91,92]), humans might have evolved a soft limit on recursive mental time travel that is ‘just right’ to support adaptive decision-making.

## 4. Links with other recursive capacities

The recursive structure of mental time travel is not only informative in and of itself, but also in that it might reflect a more general recursive property of human cognition. On this note, it is again critical to distinguish between tail recursion and nested recursion, which have distinct structures and are possibly substantiated by unrelated cognitive mechanisms. Many human cognitive capacities can evince tail recursive structure [31], including language (when combining words, phrases and sentences [93,94]), counting (when repeatedly adding 1 to a total score [95]), spatial reasoning (when mentally zooming out from a house, to a street, to a city, to a state, to a country [96]) and indeed, mental time travel (when imagining whatever, wherever, whenever [15]). As demonstrated earlier, however, online processing of tail recursive structure is not bounded by the total number of elements in a series, whereas online processing of nested recursive structure is so bounded.

In computer science, procedures with tail recursive structure can be converted to functionally equivalent but more efficient procedures with *iterative* structure, such that the computer can process each line of code sequentially without having to hold the previous line of code in memory [97]. Accordingly, some linguists have suggested that humans typically generate and process language with tail recursive structure in a similarly efficient iterative manner [98], such that truly recursive cognition might only be elicited (if at all) by language with nested recursive structure. However, because sentences with nested recursive structure are rare in human writing and even rarer in human speech [26], such perspectives suggest that a capacity for recursive thinking may not be strictly necessary for generating and processing human language ([19,20,26,98–100]; contra [31]). Mental time travel with nested recursive structure, by contrast, is routine for humans, regardless of whether one draws the line for this structure at level 1 or level 2. Indeed, as Corballis [13] suggested, the rare and relatively rudimentary instances of nested recursion in language might be mere by-products of a more general human capacity for nested recursive thinking that originally evolved to support mental time travel.

Besides mental time travel, the other obvious candidate for an evolutionary driver of nested recursive thinking is theory of mind, which has an analogous hierarchical structure of minds within minds [1,2,11,12,101]. Arguably, level 0 expressions of theory of mind include implicit belief tracking, whereby infants [102], children [103], adults [104] and non-human animals [105] appear to predict the belief-driven actions of another agent without necessarily reasoning about or even being conscious of the very beliefs driving those actions [104,106–108]. Level 1 theory of mind, by contrast—if taken to be recursive—would encompass any situation in which one possesses the metarepresentational understanding that one’s own mind is representing another mind in the form of {me{other}}, regardless of whether the represented content of that other mind is congruent or incongruent with the content of one’s own mind.^3^ Again, higher levels of theory of mind are more unambiguously recursive, as per second-order [111] and third-order [112] belief understanding. Level 3 theory of mind, for instance, can take the form of {me{other{me{other}}}}, as when you recognize that another person thinks (level 3) that you have a false belief (level 2) about what that person knows (level 1). It can also take forms such as {me{other3{other2{other1}}}}, as when you recognize what person 3 thinks (level 3) about what person 2 thinks (level 2) about what person 1 desires (level 1).

Given their similarities in nested recursive structure, it seems reasonable to suppose that mental time travel and theory of mind might draw on the same general capacity for nested recursive thinking [1]. This perspective is nonetheless complicated, however, by the fact that mental time travel and theory of mind each also draw upon distinct non-recursive processes such as those related to thinking about the self versus others [2,113]. Such distinct processes might explain the inconsistent correlations between measures of theory of mind and mental time travel (including counterfactual thinking) across developmental [114,115], clinical [116,117] and neurocognitive [118,119] domains. To my knowledge, only one (unpublished) [120] study has measured multiple levels of both theory of mind and mental time travel in a single sample. Testing a cross-section of 3- to 8-year-old children, Schidelko *et al*. [120] found that measures of level 1–3 mental time travel closely scaled with development—such that children tended to succeed on level 1 measures before level 2 measures, and level 2 measures before level 3 measures—with the size of the scalability coefficient (0.72) even greater than that for the more well-established level 1–3 theory of mind measures (0.66). Yet, this study also found only limited correlations between the mental time travel and theory of mind measures of equivalent levels. Future studies with a longitudinal design may be necessary to test the hypothesis that, across both mental time travel and theory of mind, the acquisition of common lower-level representational abilities is a precursor for acquiring common higher-level abilities.

Another apparent feature of recursive mental time travel and theory of mind is that it is feasible, and perhaps common, to engage these processes concurrently. Level 3 mental time travel, for instance, appears to be readily combined with level 3 theory of mind, as when you might anticipate (level 3) that you would immediately regret (level 2) correcting a friend’s minor misconception (level 1), for fear that she might form the false belief (level 3) that you think (level 2) she knows little about the subject (level 1). Although the total number of recursive relations entailed in this example is six (if one accepts level 1 thinking as recursive), the maximum recursive depth is only three. The apparent ease of processing such combined instances—as compared to processing standalone mental time travel and theory of mind instances with a recursive depth of six—suggests that the limits of recursive thinking may lie in maximum recursive depth rather than in the total number of recursive relations entertained *per se*. The seeming lack of interference between higher-level mental time travel and theory of mind also suggests that, if these processes do indeed draw on the same general mechanism for nested recursive thinking, then that mechanism might concurrently distribute resources across parallel neurocognitive networks (see [119]) without any cumulative strain on overall cognitive bandwidth.

Of course, both (i) the inconsistent correlations between mental time travel and theory of mind measures, and (ii) the apparent lack of interference between these two processes, might instead indicate that they do not draw on the same general capacity for nested recursive thinking after all. Accepting that premise, however, seems to necessitate the conclusion that humans have evolved (biologically or culturally [121]) two distinct and specialized mechanisms for mentally nesting minds within minds, with these two mechanisms nonetheless able to make concurrent and coherent contributions to the construction and processing of mental scenarios.

## 5. Final reflections and future directions

Here, I have outlined a preliminary framework of the recursive grammar of mental time travel. This framework distinguishes between mental time travel processes with tail recursive and nested recursive structure, delineates numerous non-recursive and recursive forms of temporal thought across representational levels, and introduces a notation by which to identify and categorize the recursive forms. There are, however, many open questions, including—to reiterate an earlier point—the fundamental ontological validity of formulating mental time travel in recursive terms.

It seems plausible, for instance, that people can deploy lower-level heuristics to similar ends as the higher-level recursive processes I have outlined. When you anticipate regret, do you necessarily anticipate your future self looking backward at looking forward (a level 3 process), or might you simply anticipate feeling bad in the future (a level 1 process)—or even just know that you would feel bad without really mentally travelling in time at all (a semantic process)? One possibility is that such lower-level heuristics are indeed dominant over higher-level recursive processes, and yet they only become dominant via automatization after lots of practice at the higher-level processes during development (see [122] for an analogous argument about theory of mind). Another possibility, however, is that lower-level processes have primacy in human mental time travel (and theory of mind), and the recursive grammar is simply a means to categorize the outputs of these processes rather than necessarily reflecting recursive inputs *per se*. Nonetheless, just as recursive models of language [93,94] have galvanized research and theorizing in linguistics and cognitive science more broadly, it is clear that a recursive model of mental time travel can spur new ways of conceptualizing and studying temporal cognition—even if that recursive model itself is ultimately *just* a model.

For a start, the current recursive framework makes clear predictions about the developmental trajectory and phylogenetic distribution of temporal cognition. In particular, it suggests that children and animals should never be capable of nominally higher-level forms of recursive mental time travel without also being capable of nominally lower-level forms (at least when holding multiplicity constant). In children, at least some expressions of level 1–3 mental time travel do appear to follow such a developmental trajectory, in that children first show compelling evidence of {present{futures}} representations around 4 years of age, and then {present{past{futures}}} representations around 6 years of age, and finally {present{futures{past{futures}}}} representations around 8 years of age [10,11]. One general challenge for research with young children and animals, however, is to design experiments that can distinguish between: (i) behaviour that is genuinely underpinned by mental time travel processes with recursive structure, and (ii) behaviour that is instead underpinned by pseudo mental time travel processes such as those outlined in the section on level 0 temporal thought. This will not be an easy challenge, as foreshadowed by related ongoing debates about whether very young children [17,59,123–126] and non-human primates [11,59,127–131] can represent mutually exclusive futures in the integrated form of {present{futures}}. Nonetheless, if the addition of nested recursive structure to temporal cognition is indeed meaningful in an evolutionary sense, then it must be tractable to natural selection and thus also somehow tractable to experimental inquiry.

On a more procedural note, the bracketed notation I have introduced here is illustrative but underdeveloped, in that it calls attention to the key aspects of the representational hierarchy under consideration while also lacking precision. The notation {present{past{future}}}, for instance, does not specify whether the relative future (from the perspective of the past) is situated in the absolute past, present or future. A more precise notation might incorporate positive and negative subscripts to denote absolute time (with present time set at 0), such that {present_0_{past_-1_{future_1_}}} would indicate that the relative future is in the absolute future, {present_0_{past_-1_{future_0_}}} would indicate that the relative future is in the absolute present, and {present_0_{past_-2_{future_-1_}}} would indicate that the relative future is in the absolute past. The illustrative notation also does not distinguish between the total number of possible futures under consideration for a future-oriented perspective with multiplicity. This aspect might be denoted with superscripts, such that {present_0_{past_-1_{futures_0_}^2^}} would indicate counterfactual thinking with two represented possibilities, whereas {present_0_{past_-1_{futures_0_}^3^}} would indicate counterfactual thinking with three represented possibilities (e.g. ‘it could have been *better* than it *is*, but it also could have been *worse*’). Future research might examine how such distinctions are reflected in cognition, emotion and behaviour, in addition to the primary distinctions of recursiveness, multiplicity and relative temporal structure.

The concept of recursion appears to hold much promise for furthering our understanding of complex and perhaps uniquely human cognition, well beyond its traditional applications in linguistics [93,94]. However, whereas other influential frameworks have proposed a broader role of recursion in producing discrete infinity [2,31], the current framework emphasizes that more finite expressions of recursion might have been similarly important in making humans who we are. We might not be able to endlessly embed minds within minds across different times, but the limited temporal depths that we can traverse might have been just right for opening up new frontiers of thought and behaviour.

## Data Availability

This article has no additional data.

## Footnotes

^1^
Karlsson [26] more formally defines nested recursion as when ‘A nested structure X is embedded within a structure of the same type, X^n−1^, with non-null material ... of X^n−1^ both to the left and to the right of X^n^’ (p. 51).

^2^
One apparent exception to this general principle is in cases of epistemic uncertainty [62], where a past event has unfolded in a particular manner and yet it is unclear just how (e.g. when comparing two contradictory witness accounts of a crime). As pointed out by an anonymous reviewer, however, one might typically represent such past alternatives by first mentally travelling back to a point in time *before* the past event and then travelling forward towards the relevant temporal juncture (i.e. level 2 mental time travel). Alternatively, one might typically represent such past alternatives in the form of level 1 future alternatives, given that one’s *knowledge* of what actually happened can only be gained at some point in the future [11].

^3^
This formulation entails a distinction between: (i) the notion of metarepresentation involved in *representational nesting* (emphasized herein) and (ii) the notion of metarepresentation as *representing the representational relation* (denoted by Pylyshyn [109] and elaborated on by Perner [110]). Whereas the first notion encompasses the recursive structure of multiple representations hierarchically organized across higher and lower levels, the second notion encompasses knowledge of what a representation *is* (at any given level). On this view, any added difficulty with representing another’s false belief compared to, say, representing another’s true belief or desire, is not a function of added complexity in representational nesting, but rather a function of the added necessity of representing the representational relation at level 1. Also, given that representing the representational relation often entails holding two contradictory representations in mind (true and false), this form of metarepresentation is more reflective of the current notion of *multiplicity* than the current notion of recursion (also see [11]). I am grateful to an anonymous reviewer for helping me to recognize and clarify this distinction.
